# Sub-cone visual acuity can be achieved with less than 1 arcmin retinal slip

**DOI:** 10.1167/jov.26.2.7

**Published:** 2026-02-13

**Authors:** Veronika Lukyanova, Julius Ameln, Jenny L. Witten, Aleksandr Gutnikov, Maximilian Freiberg, Bilge Sayim, Wolf Harmening

**Affiliations:** 1 Department of Ophthalmology, University of Bonn, Bonn, Germany; 2 Zeiss Vision Science Lab, Institute for Ophthalmic Research, Tübingen, Germany; 3 Berliner Hochschule für Technik, Berlin, Germany; 4 Laboratoire de Sciences Cognitives et Psycholinguistique (LSCP), Département d’Études Cognitives (DEC), École normale supérieure (ENS), UMR8554, Centre National de la Recherche Scientifique (CNRS), Paris, France

**Keywords:** foveal vision, adaptive optics, micro-psychophysics, fixational drift, cone photoreceptors

## Abstract

The retinal area inspecting a visual stimulus and, consequently, the number of photoreceptors engaged in a visual task increase with presentation time, as fixational eye movements continuously move the retina across the retinal image. Here, we varied stimulus duration in a Tumbling-E visual acuity task while recording videos of the photoreceptor mosaic in seven participants with adaptive optics micro-psychophysical techniques to determine how far the retinal image must move across the cone mosaic before this motion begins to improve visual acuity. Five stimulus presentation durations were tested (3, 80, 220, 370, and 600 ms), while participants exhibited natural eye movements. Retinal slip amplitudes (i.e., the total displacement stimuli underwent) increased linearly with stimulus duration at individual rates. Higher cone density was associated with drift over smaller retinal areas, making the number of traversed cones more similar across participants at longer durations. At the shortest presentation duration, retinal slip was virtually absent and acuity was limited by retinal resolution, averaging to 1.07 ± 0.08 times the cone row-to-row spacing (Nyquist limit of sampling). At an 80-ms duration, corresponding to approximately two cone diameters of retinal slip, acuity thresholds improved significantly, reaching 0.90 ± 0.10 of the Nyquist limit. Thresholds continued to improve with longer durations at a lower rate, reaching 0.75 ± 0.10 times the Nyquist limit at 600 ms. These results demonstrate that humans can extract visual information with sub-cone precision within less than 100 ms, with a retinal slip approaching single foveal cone spacing.

## Introduction

When humans fixate on a visual object, incessant fixational eye movements (FEMs) translate retinal photoreceptors across the retinal image, dynamically updating visual sampling (Dodge, 1907; Yarbus, 1967; Martinez-Conde, Macknik, & Hubel, 2004). This constant retinal slip creates a link between spatial sampling and the temporal exposure to a stimulus. One consequence is that more information is potentially yielded with longer fixation. Here we ask how many foveal cones a stimulus has to traverse to benefit visual acuity.

In the absence of any motion, our ability to resolve fine detail is theoretically limited by both the quality of the retinal image and by the sampling limit of the neural machinery (Campbell & Green, 1965; Westheimer, 2009). In the center of the foveola, the central 1-degree diameter of the retina, cone photoreceptor density is highest and the ascending visual pathways are built to preserve the cones’ spatial grain (Walls, 1942; Polyak, 1957; Curcio & Allen, 1990; Tuten & Harmening, 2021). Under optimal optical conditions, when diffraction sets the upper bound to the quality of the retinal image, foveolar cone spacing dictates the highest resolvable spatial frequency before aliasing occurs (Westheimer & McKee, 1975; Williams, 1985). Thus, maximum visual resolution ought to be capped at the Nyquist limit of cone sampling, which equals the smallest row-to-row spacing of the foveal mosaic. By compensating for the eyes’ natural aberrations with adaptive optics-corrected stimuli presented during natural FEM, however, visual acuity was shown to exceed this limit, reaching values as high as 20/8 vision, corresponding to spatial details that are 20% smaller than the Nyquist limit (Rossi, Weiser, Tarrant, & Roorda, 2007; Witten, Lukyanova, & Harmening, 2024). It is likely that the visual system leverages the temporal modulations in cone activity as produced by FEM to increase resolution beyond static sampling limits (Pitkow, Sompolinsky, & Meister, 2007; Ahissar & Arieli, 2012; Nghiem, Witten, Dufour, Harmening, & Azeredo da Silveira, 2025).

Fixational drift, characterized by slow, small-amplitude movements, was shown to be exploited by the visual brain in acuity tasks by its main feature—continuous motion—which leads to constant refresh of the visual input (Rucci & Poletti, 2015). Drift motion patterns are often described by random-walk statistics in theoretical models (Pitkow et al., 2007; Burak, Rokni, Meister, & Sompolinsky, 2010; Engbert, Mergenthaler, Sinn, & Pikovsky, 2011; Kuang, Poletti, Victor, & Rucci, 2012; Anderson, Ratnam, Roorda, & Olshausen, 2020) and experimental research (Nachmias, 1961; Kuang et al., 2012; Intoy & Rucci, 2020; Clark, Intoy, Rucci, & Poletti, 2022; Ben-Shushan, Shaham, Joshua, & Burak, 2022), but also as more structured, nonrandom patterns (Malevich, Buonocore, & Hafed, 2020; Hafed, Chen, Tian, Baumann, & Zhang, 2021). This indicates that drift could be not only exploited but also controlled by the visual system in a favorable way, such as by moving retinal areas of higher cone density toward the object of interest (Witten et al., 2024). On a mechanistic level, drift may enhance acuity through optimal spatiotemporal flow of the retinal image either through sensor-derived temporal encoding (Ahissar & Arieli, 2001) or luminance modulations (Rucci & Victor, 2015). Moreover, the ongoing movement provides not just singular snapshots but multiple views of the retinal image (Ratnam, Domdei, Harmening, & Roorda, 2017; Anderson et al., 2020). At the same time, drift introduces spatial noise, posing a challenge that the visual system must compensate for (Packer & Williams, 1992; Murakami & Cavanagh, 1998; Murakami & Cavanagh, 2001; Pitkow et al., 2007; Burak et al., 2010). This might be achieved by neural stimulus tracking if minimal a priori knowledge of the stimulus is present (Nghiem et al., 2025).

Testing fixational drift as a mechanism that potentially aids acuity can be explored by varying the extent of retinal slip it produces. Such manipulation can be achieved by either retina-contingent stimulation (stabilization) (Ditchburn & Ginsborg, 1952; Riggs, Ratliff, Cornsweet, & Cornsweet, 1953; Pritchard, 1961; Heckenmueller, 1965; Yarbus, 1967; Stevens et al., 1976; Kelly, 1979; Hammer et al., 2006; Arathorn et al., 2007) or control of stimulus exposure duration (Riggs et al., 1953; Tulunay-Keesey & Jones, 1976). While early studies suggested better or no changes in performance under stabilization (Riggs et al., 1953; Keesey, 1960; Tulunay-Keesey & Jones, 1976; Kelly, 1979), more recent work using modern instrumentation indicates that external retinal stabilization degrades the perception of fine spatial detail (Rucci, Iovin, Poletti, & Santini, 2007; Ratnam et al., 2017; Anderson et al., 2020; Intoy & Rucci, 2020). Experiments that manipulated presentation duration have shown that acuity generally improves with increasing stimulus exposure, plateauing after a few hundred milliseconds (Baron & Westheimer, 1973; Tulunay-Keesey & Jones, 1976; Alexander, Derlacki, Fishman, & Szlyk, 1993; Niwa & Tokoro, 1997; McAnany, 2014), or, in some cases, continues to improve up to 10 seconds (Heinrich, Krüger, & Bach, 2010). Most psychophysical studies that measure acuity typically use stimulus durations of 500 ms or longer to ensure saturated performance. During this time, the retinal image moves across a space equivalent to 30–50 foveal cone diameters (Rolfs, 2009; Ameln et al., 2025), an order of magnitude above the sampling limit. This leaves the minimal number of cones a stimulus must traverse to produce a measurable improvement in acuity not yet established by previous work.

Given the spatiotemporal interaction that FEM exerts on cone sampling, we investigated how visual acuity relates to stimulus duration and asked what the minimum retinal slip is that produces a measurable benefit to visual acuity. To disentangle the contributions of retinal resolution and eye movement, we used an adaptive optics scanning light ophthalmoscope (AOSLO). The AOSLO corrects the eye's higher-order optical aberrations (Roorda et al., 2002), produces cell-resolved images of the foveola with unambiguous landing positions of retinal stimuli (Reiniger, Domdei, Holz, & Harmening, 2021), and allows precise tracking of retinal motion (Arathorn et al., 2007; Stevenson & Roorda, 2005). Thus, we ensured that any observed performance changes were driven by the interplay between FEM, stimulus duration, and cone topography, rather than optical aberrations.

## Methods

### Participants

Seven human observers (three males and four females, mean age: 29.6, range: 19–44 years) with no known eye disease participated in the experiment. Written informed consent was obtained from all participants in accordance with the Declaration of Helsinki. The study was approved by the independent ethics committee of the Rheinische Friedrich-Wilhelms-Universität Bonn. General eye health was confirmed by an ophthalmologist. Pupils were dilated, and accommodation was paralyzed by administration of two drops of 0.5% tropicamide 15 minutes before the experimental session, with additional drops administered if necessary to ensure adequate mydriasis and cycloplegia throughout the experiments. Imaging and psychophysical testing were conducted in the dominant eye only, identified using the Miles Test prior to dilation (right eyes in all participants). Participants’ refractive errors by means of spherical equivalent ranged from plano to −1.0 diopter. To position and stabilize the head in front of the imaging instrument, a custom dental impression (bite bar) was made for each participant. Participant naming used throughout the analysis (P1–P7) followed an ascending order of their cone density at the anatomical center of the foveola, expressed in cones per square degree of visual angle.

### AOSLO Micro-Psychophysics

**Figure 1.**
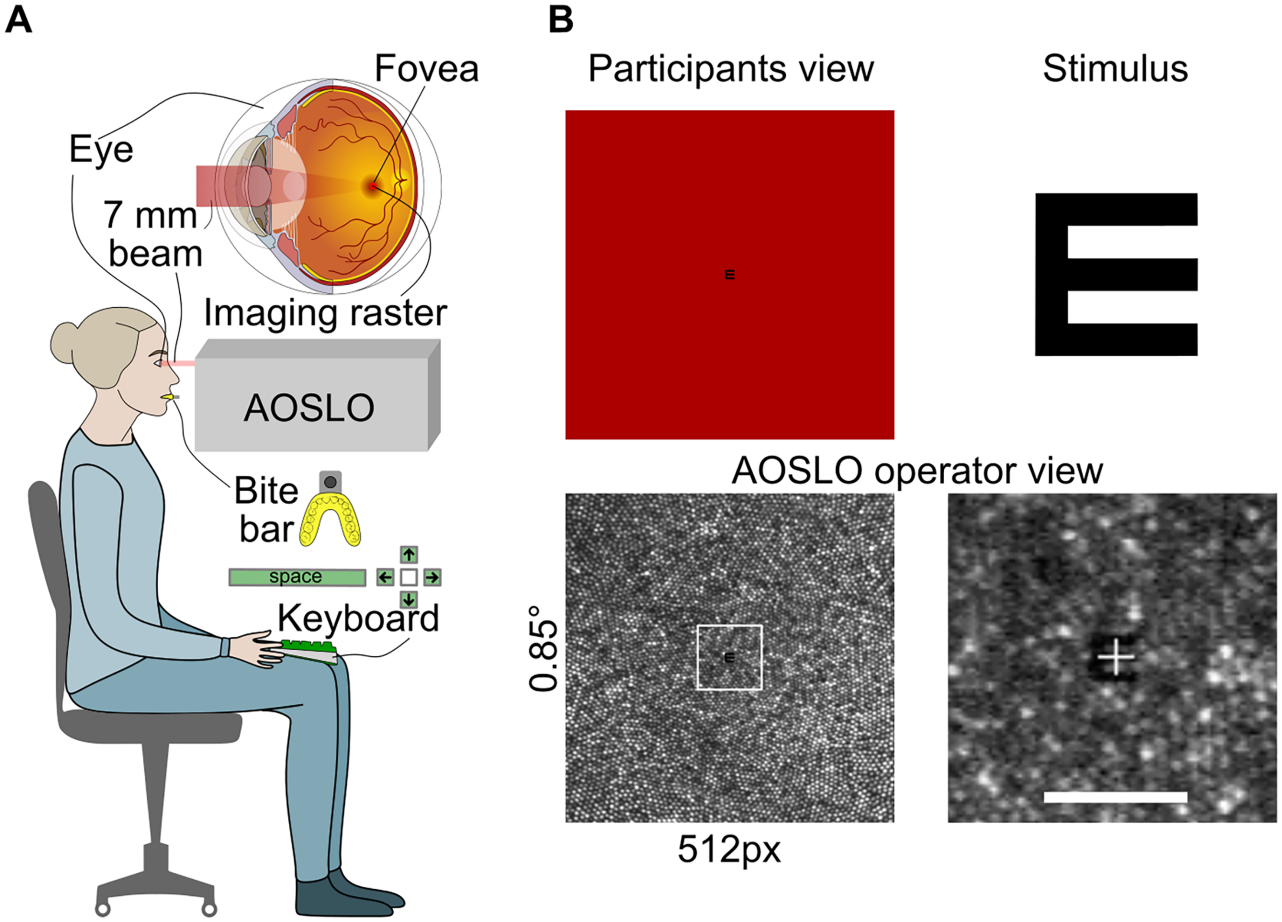
High-resolution AOSLO imaging and micro-psychophysics. (**A**) Schematic representation of the setup for foveal acuity testing with AOSLO. Participants sat upright in the AOSLO system, with head movements quelled by a custom-made bite bar. A 788-nm, 7-mm diameter beam was directed into the participant's eye and scanned across a 0.85-degree field. Each trial was initiated by the participant pressing the spacebar on a keyboard placed on their lap, triggering the recording of a 1-second AOSLO video. This was followed by reporting the orientation of the Tumbling-E stimulus using one of the arrow keys. (**B**) The Tumbling-E acuity stimulus, shown in the top right panel, appeared in the center of the scanning raster (top left). The AOSLO operator concurrently observed the retinal image, visualizing the participant's cone mosaic (lower left). The bottom right panel shows a magnified view of a single AOSLO video frame with the stimulus visible at the center. Scale bar: 5 arcmin.

For in vivo retinal imaging and visual stimulation with foveal cone resolution, a custom-built AOSLO was used. Instrument details and micro-psychophysical procedures have been described before (Roorda et al., 2002; Domdei, Reiniger, Holz, & Harmening, 2021). In short, the AOSLO created an image of and a stimulus on the retina of the test eye by an intensity-modulated point-scanned 788-nm light, spanning a square field on the retina of 0.85 × 0.85 degrees of visual angle (Figure 1). Ocular aberrations were compensated by closed-loop adaptive optics correction, ensuring continuous diffraction-limited beam formation for both imaging and stimulation, irrespective of experiment duration. The AOSLO creates videos from which the exact location and motion path of a retinal stimulus can be assessed with high temporal and subcellular spatial resolution by image registration techniques.

Prior to the first experimental sessions, a high-resolution foveal montage was created for each eye, similar to that previously described (Ameln et al., 2025). At least three videos were recorded for 10 fixation locations, including the center, corners, and border midpoints of the imaging raster. Videos were stabilized offline using an improved strip-wise image registration technique based on an earlier implementation (Stevenson, Roorda, & Kumar, 2010). The images were combined into a roughly 1.5 × 1.5-degree foveal montage using both custom automontaging software (Chen et al., 2016) and manual blending in Corel Photo-Paint (CorelDRAW Graphics Suite 2019; Alludo, Ottawa, Canada) to reduce residual image distortions. In such montages, all cone center locations were annotated using ConeMapper, a custom neural network–assisted MATLAB tool for identifying cone locations (Gutnikov et al., 2025), followed by manual verification and correction. Cone density maps were generated via Voronoi diagrams by averaging the area of the 150 closest cones to each pixel in the map. The cone density centroid (CDC), representing the anatomical foveal center, was determined as the weighted center of the top 20% cone density contour (Reiniger et al., 2021). The average distance to neighboring cones (intercone distance, ICD) was computed for every cone in the montage and employed for a trial-based estimation of each individual cone Nyquist limit (N_c_) by N_c_ = ICD × (√3)/2.

### Stimuli and procedure

Visual acuity was assessed in a four-alternative forced-choice orientation discrimination of a Tumbling-E optotype (Figure 1B). Throughout this article, we define the stimulus size as the stroke width of the E. The stroke width corresponds to one fifth of the full height of the optotype and is equal to the gap width between the limbs of the E. Orientation was varied pseudo-randomly and chosen from one of the four cardinal orientations (up, down, left, right) for each trial. Stimuli were computationally constructed as bitmaps with a bit depth close to 10 bits (1,000 gray values). To achieve subpixel stimulus resolution, a Gaussian filter with a kernel size of 5 pixels and a sigma of 1 pixel was applied to the nominal stimulus (Guizar, 2025) before it was computationally resized to the desired value. To avoid border artifacts, stimuli were sufficiently zero-padded. A single stimulus presentation was initiated by the participant by a keyboard button press. After presentation, perceived orientation was reported using one of the four arrow keys on the keyboard (Figure 1A).

**Figure 2.**
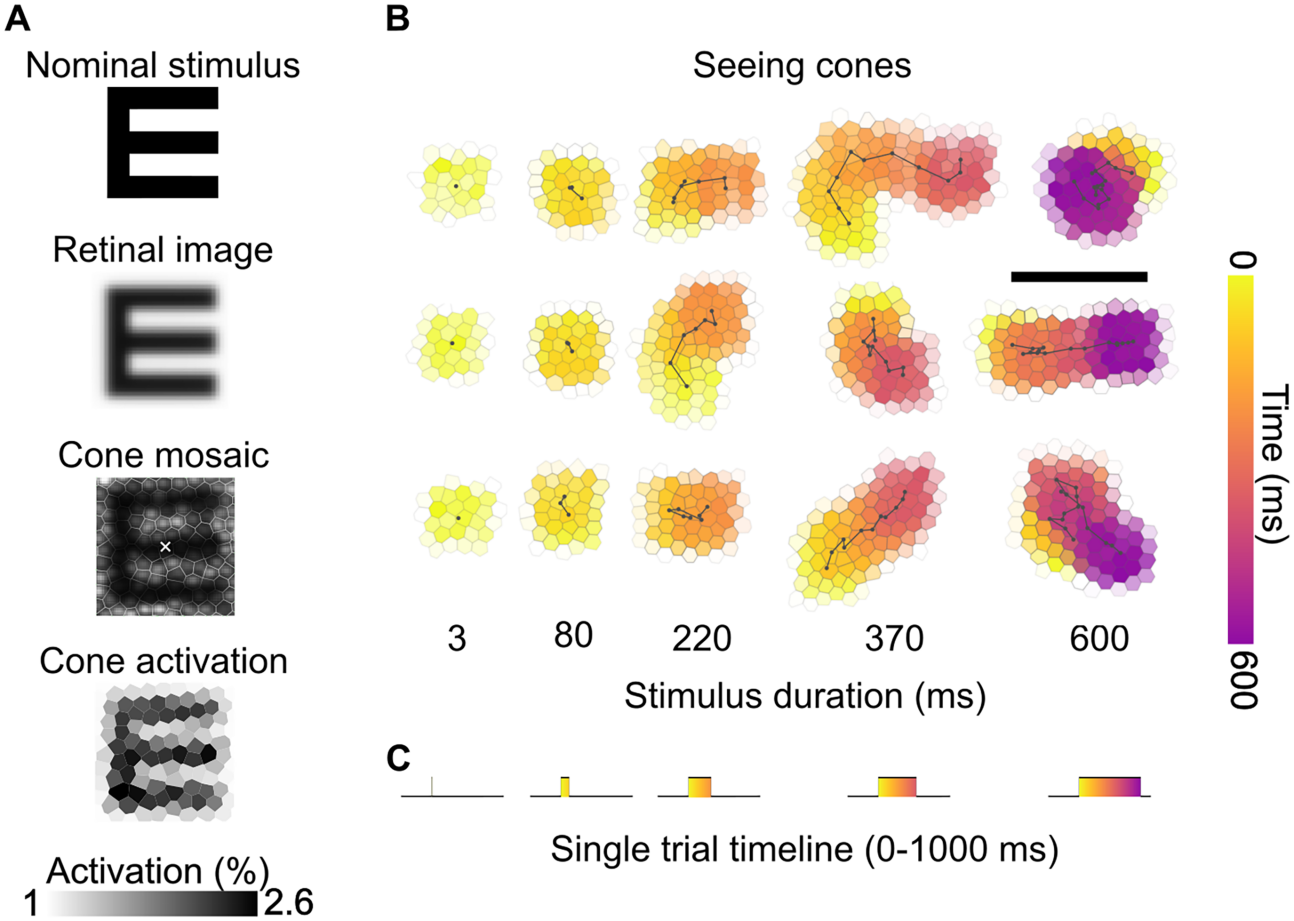
Cone activation and seeing cones across stimulus durations. (**A**) Computation of single cone activation: The nominal stimulus is blurred by the eye's optics. Multiplication of the retinal stimulus with the underlying cone mosaic light apertures results in a cone activation map. Because the E was presented in OFF contrast (a dark E on a red background), cones receiving less light show higher activation and therefore appear darker. The grayscale represents normalized activation (percentage of maximum cone activation). (**B**) Seeing cones were determined by cone activation patterns over the course of exhibited drift trajectories. Three example trials (participant P2) are shown at each stimulus duration. Each hexagonal cell represents a cone; the color indicates the time course of stimulus motion across the cone mosaic (yellow = early, purple = late). Black lines show the fixational drift trajectory during stimulus presentation. As duration increases, retinal slip covers progressively larger portions of the cone mosaic. Scale bar: 5 arcmin. (**C**) Single-trial timeline, where a 1-second video is recorded. The stimulus onset occurs at approximately 300 ms and is presented for a variable duration, ranging from 3 to 600 ms.

Stimulus onset was during the eighth frame after trial initiation (i.e., after ∼300 ms) and presented for 1, 3, 7, 11, or 16 AOSLO frames, which corresponds to a duration of approximately 3, 80, 220, 370, and 600 ms (Figures 2B, 2C). In our AOSLO system, one video frame is composed of 512 lines, each sampled with 512 pixels, and is captured approximately every 37 ms (frame rate: 27 Hz). Stimuli are produced by turning the light source briefly off by acousto-optic modulation at appropriate times, corresponding to the pixel space. Most stimulus sizes were very small; the largest stimulus had a stroke width of 8 pixels (equaling 48 seconds of arc of visual angle, arcsec) and thus occupied less than 13% of the horizontal and vertical dimensions of the raster. It took approximately 3 ms for the laser to sweep across an area defined by such stimulus geometry (from top left to bottom right pixel). Despite the frame rate, stimuli spanning multiple frames were perceived by the observers as continuous and not flickering. Stimulus duration was thus defined as the time from when it first appeared to when it was switched off in the last frame. All stimuli were drawn at the center of the raster.

For each duration condition, an acuity threshold was determined in at least five repeated runs, with 23 trials per run. Stimulus size in each trial followed an adaptive staircase. Initial stimulus size was set to 48 arcsec. After each correct response, stimulus size was reduced by a factor of 1.75 until the first incorrect response, indicating the approximate region of the presumed threshold. From there, a two-down, one-up rule with 1.5 step size up and 0.82 step size down was applied (García-Pérez, 1998). Every sixth trial was a motivational stimulus where the stimulus was set to 48 arcsec (Bach, 1996). Before the experimental session, participants did five test runs each consisting of 23 trials with a 500-ms presentation duration to become acquainted with the testing procedure.

Each dataset underwent curation prior to psychophysical analysis, during which, on average, 30% of all recorded trials were removed from the analysis. Trials were excluded if saccades, microsaccades, or blinks occurred during stimulus presentation. Additionally, trials were removed if technical issues disrupted stimulus presentation, such as missing stimulus features or altered stimulus appearance on the retina, both possible artifacts of the stimulus delivery hardware. Trial elimination was performed using custom-written software that identified the time periods during which stimuli were presented. It calculated eye movement velocity within those intervals and flagged trials where it exceeded 30 arcmin/s (indicating a microsaccade). Cross-correlation was used to compare the intended stimulus geometry with the stimulus as presented on the retina, allowing detection of distorted or missing presentations. Results of this procedure were verified by a human observer by inspecting each case visually. Trials across repeated runs were pooled and binned to yield at least seven representative bins. Bin sizes varied depending on the available stimulus sizes, with widths ranging from 5 to 11 arcsec, and were used to compute psychometric function fits. The visual acuity threshold, defined as the stimulus size required for 62.5% correct responses, was estimated by fitting the pooled data to a Weibull distribution function using the MATLAB toolbox Psignifit (Wichmann & Hill, 2001). In general, lower threshold values indicate better acuity.

### Ocular drift analysis

Eye motion traces were extracted from the 1-second AOSLO videos by strip-wise image registration with a temporal resolution of 864 Hz (Stevenson & Roorda, 2005). Because of registration artifacts that are due to reference frame distortions and ocular torsion (Hofmann, Domdei, Jainta, & Harmening, 2022), high-resolution motion traces were down-sampled by linear interpolation between the central samples in each frame. Retinal slip during stimulus presentation was quantified by the total slip exhibited, calculated as the sum of the concatenated drift motion vector lengths. To quantify drift variance, we first computed the mean squared displacement (MSD) for each trial. Then, at each time point, the variance across all MSD curves from repeated presentations was calculated. This yielded a time-dependent measure of how drift dispersion evolved across trials. The drift-variance value reported corresponds to the variance at the 600-ms time point.

To better understand the role of cone photoreceptors directly involved when a stimulus is presented to the retina, we introduce a metric termed *seeing cones* (Figure 2A). Unique seeing cones per trial were found by examining all AOSLO video frames where the stimulus appeared. Subsequently, we registered these frames to an annotated cone montage to determine which cones were covered by the stimulus. We then applied a simple model of light capture, assigning each cone a light acceptance aperture, with its diameter estimated as 48% of the average spacing between neighboring cones, using a Gaussian approximation (Macleod, Williams, & Makous, 1992). The retinal image was computed by convolving the eye's diffraction-limited point spread function (calculated for 788 nm light and a 7-mm pupil) with the nominal stimulus. The retinal image was overlaid onto the cone aperture model, and both matrices were multiplied. The total light capture was calculated for each trial throughout all the frames when the stimulus was presented, and the percentage of light captured by each cone was determined. Cones capturing more than 1% of the total light—corresponding to the smallest detectable contrast (Fechner, 1966; Pelli & Bex, 2013)—were classified as *seeing cones*.

In our simplified model, we exclude considerations of a cone's temporal decay function. In AOSLO-based stimulus delivery, the stimulus is projected onto the retina by modulating the scanning laser's intensity, specifically by switching it off to deliver light decrements relative to the scanning raster as the laser traverses the retina (Poonja, Patel, Henry, & Roorda, 2005). Consequently, each retinal location within the scanning raster, excluding the stimulus delivery area, receives a single brief pulse of focused light within each frame cycle (approximately every 37 ms in our system if no movement occurred). We interpret the light decrements defining the stimulus as activation signals, based on the presence of equally distributed ON and OFF visual pathways in the foveola (Polyak, 1957). Even though a functional asymmetry in activation between those pathways has been shown, we assumed that acuity performance is likely unaffected by such asymmetries (Chichilnisky & Kalmar, 2002; Patterson, Cai, Yang, Merigan, & Williams, 2025).

In the condition where stimuli were presented for a single frame (a 3-ms duration), we assumed the slip to be zero for the seeing cone calculation. This assumption likely holds: Based on the observed average drift velocity of about 13 arcmin/s, a 3-ms duration would equate to less than 2.4 arcsec of exhibited slip, a displacement of less than a tenth of a single cone diameter on the retina.

## Results

**Figure 3.**
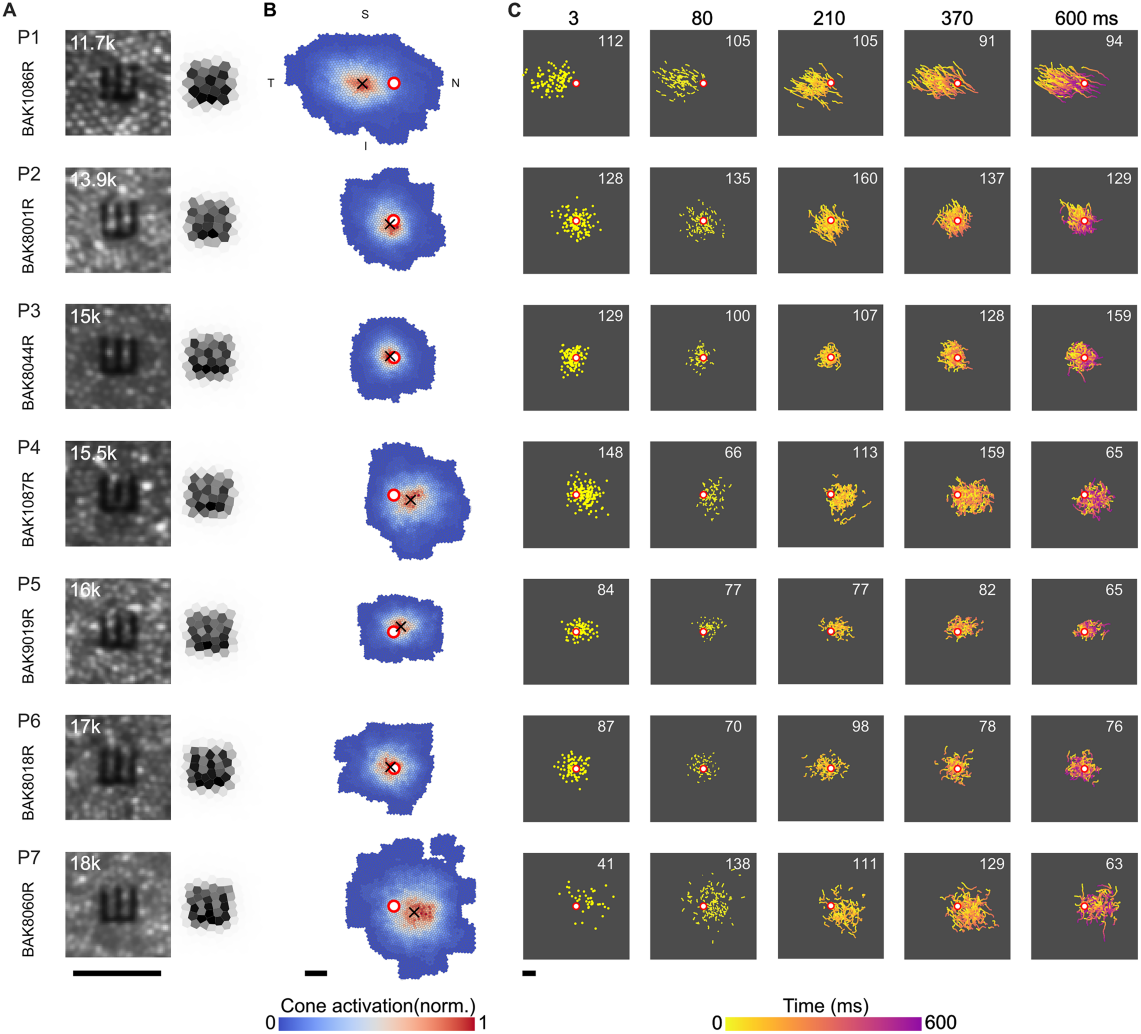
Retinal sampling and motion traces. (**A**) AOSLO image crops for all participants (P1–P7, rows) centered on the CDC, with a 25-arcsec Tumbling-E stimulus superimposed. The panels on the right show the corresponding cone activation maps. Each hexagonal element represents an individual cone, and the grayscale value indicates the relative activation of that cone during a single frame. Cone density in cones/deg^2^ at the CDC is indicated in the upper left corner of each AOSLO image. (**B**) Seeing cone heatmaps across all trials. The CDC is indicated by the red-white circle marker. The ISOA centroid (i.e., the average stimulus location) is labeled with the black cross marker. Only right eyes were tested. (**C**) Motion traces for all trials across all participants. Columns are stimulus durations. The number shown within each panel denotes the count of valid trials. Color indicates time after stimulus onset. All scale bars are 5 arcmin.

All participants exhibited significant differences in foveal anatomy, eye movement patterns, and visual acuity, highlighting individual variability in foveal structure and function (Figure 3; Table 1).

**Table 1.** Cone mosaic and drift characteristics. *Notes:* For each participant, the table lists the cone density and corresponding Nyquist sampling limit at the CDC, mean drift velocity, drift length at 600 ms, drift variance at 600 ms (dispersion of drift trajectories), angular subtense and total number of seeing cones engaged during the task, and the offset between the CDC and the centroid of the stimulus landing region (ISOA).

Participant #	Cone density and Nyquist limit at CDC (cones/deg²)/(arcsec)	Drift velocity (arcmin/s)/(cones/s)	Drift length 600 ms (arcmin)	Drift variance (arcmin²)	Seeing cones (deg²)/(*N*)	ISOA to CDC distance (arcmin)
P1	11,749/30.9	17.5/31.2	10.2	1925	0.18/1,793	7.2
P2	13,883/28.4	11.9/23.9	6.9	321	0.11/1,422	1.2
P3	15,005/27.4	12.5/27.6	7.6	129	0.08/1,140	0.94
P4	15,466/26.9	16.1/36.2	10	98	0.13/1,677	4
P5	16,016/26.5	10/22.1	5.9	26	0.07/1,043	2
P6	17,000/25.7	10.4/23.9	6.3	40	0.1/1,476	0.86
P7	17,971/25	14/31.1	8.2	280	0.19/2,690	4.8

### Foveolar topography and retinal location

Foveal cone density, and hence sampling limits, differed markedly across participants, spanning from relatively sparse (11.8k cones/deg^2^, 31 arcsec) to dense (18k cones/deg^2^, 25 arcsec) mosaics (Figure 3A; Table 1). When a constant-size Snellen E was projected onto each retina, these differences highlighted how individual sampling limits might come into play. Because eye movement patterns also varied, the retinal area used during the acuity task differed across observers (Figure 3B). As a result, both the extent of the seeing region and the number of cones contributing to it varied (Table 1). Despite this, mostly a core subset of cones saw stimuli: Those stimulated more than 10 times accounted for 67% to 78% of all engaged cones.

Stimuli were also seen by different parts of the participants’ retinas. The average location of all stimulus presentations across all trials, defined as the centroid of the isocontour area (ISOA) of 68% of stimulus landing points, varied in distance from the CDC (Figure 3B; Table 1). The ISOA centroids for participants P1, P7, and P4 were located the farthest from the CDC, at distances of 7.2, 4.8, and 4 arcmin, respectively. In contrast, participants P2 and P5 positioned stimuli closer to the CDC, with shifts of 1.2 and 2.0 arcmin. Notably, participants P3 and P6 had ISOA centroids positioned less than 1 arcmin from the CDC.

Linear regression did not show a significant relationship between cone density at CDC and the total area covered by all seeing cones (*p* = 0.8) or the number of seeing cones (*p* = 0.51). Also, both area (*p* = 0.053) and number of seeing cones (*p* = 0.25) did not relate significantly to drift velocity. However, a significant relationship was observed between drift velocity and the distance from the ISOA centroid to the CDC: Drift velocity was higher when the distance was larger (*R*^2^ = 0.74, *p* = 0.01). Illustrations of all retinal slip trajectories for each duration condition demonstrate the individual use of retinal space over time, reflecting individual FEMs and their continuous presence throughout the visual task (Figure 3C).

### Retinal slip and seeing cones

**Figure 4.**
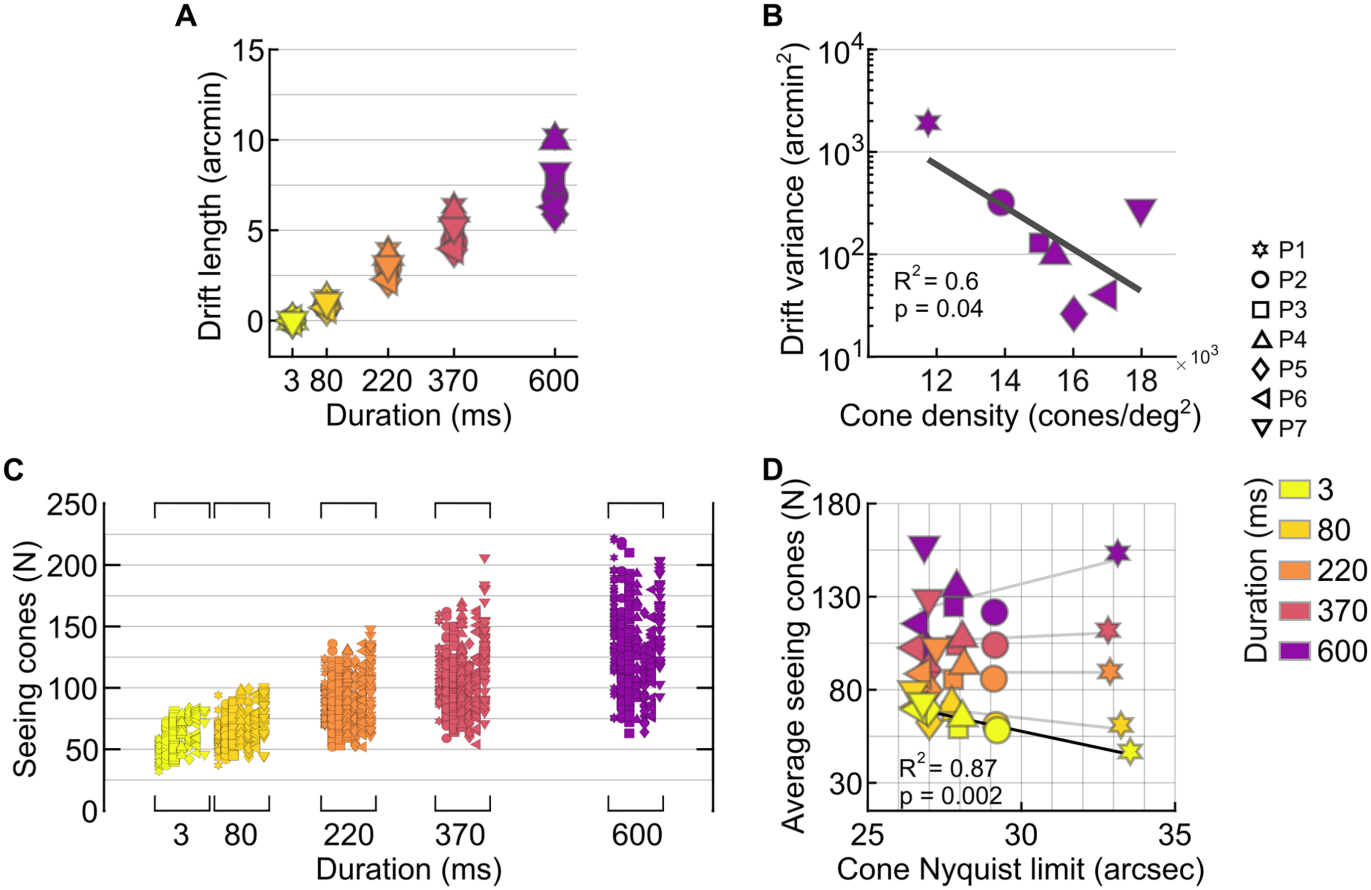
Drift characteristics and seeing cones. (**A**) Average retinal slip length for each participant (marker type) is plotted against presentation duration (color). Retinal slip grew linearly and varied among individuals, peaking at 600 ms, where it reached an average of 7.8 ± 2 arcmin, and the difference between those with the lowest and highest retinal slip reached a factor of 1.7. (**B**) Drift variance at 600 ms as a function of cone density at the CDC. The black line shows the linear regression fit. (**C**) Seeing cones plotted for individual trials where stimulus size ranged between 24 and 36 arcsec for each participant. Each data column is one participant. (**D**) Average seeing cones as a function of individual cone density, given as their Nyquist limit. Black line indicates linear regression model fits where *p* < 0.05; gray lines indicate nonsignificant correlations.

Individuals also showed substantial differences in their drift velocity, the amount of retinal distance traversed, and the temporal variability of their drift trajectories. When averaged across all trials and conditions, retinal slip velocity ranged from 10 to 17.5 arcmin/s across participants or, equivalently, 22 to 36 cone diameters per second in the individual eye (Table 1). Velocities differed significantly between observers (Kruskal–Wallis test, χ²(6) = 1,101.14, *p* < 0.001) but did not vary systematically with stimulus duration within observers (all *p* > 0.05). On average, drift length rose steadily across the four longer durations, but participants differed substantially in the magnitude of this increase. Variability was most pronounced at 600 ms, where drift lengths spanned a wide range (Figure 4A; Table 1). At this duration, drift length correlated positively with the distance between each observer's ISOA centroid and CDC (*R*^2^ = 0.67, *p* = 0.025). At 600 ms, the log-transformed drift variance showed an inverse relationship with cone density at the CDC (*R*^2^ = 0.6, *p* = 0.04): Participants with higher cone density exhibited smaller positional variance over time, whereas those with sparser mosaics displayed greater dispersion (Figure 4B; Table 1).

To examine how drift affects the spatial extent of retinal sampling, we quantified the number of cones stimulated over time on a trial-by-trial basis (Figure 4C). In principle, observers with higher cone density or faster drift would be expected to activate more cones than those with slower drift or sparser mosaics. To isolate interindividual anatomical differences, we restricted this analysis to acuity targets between 24 and 36 arcsec. For the ultra-brief 3-ms duration, the number of seeing cones increased with higher cone density (*R*^2^ = 0.87, *p* = 0.002), as expected (Figure 4D). However, for the 80- to 600-ms durations, no significant relationship was found between cone density and the number of seeing cones. Taken together with the drift-variance analysis, these results indicate that the slip exhibited by each eye partially reduced the anatomical differences in cone density across participants, contributing to a more consistent number of seeing cones at the longer durations.

### Acuity thresholds at different stimulus durations

**Figure 5.**
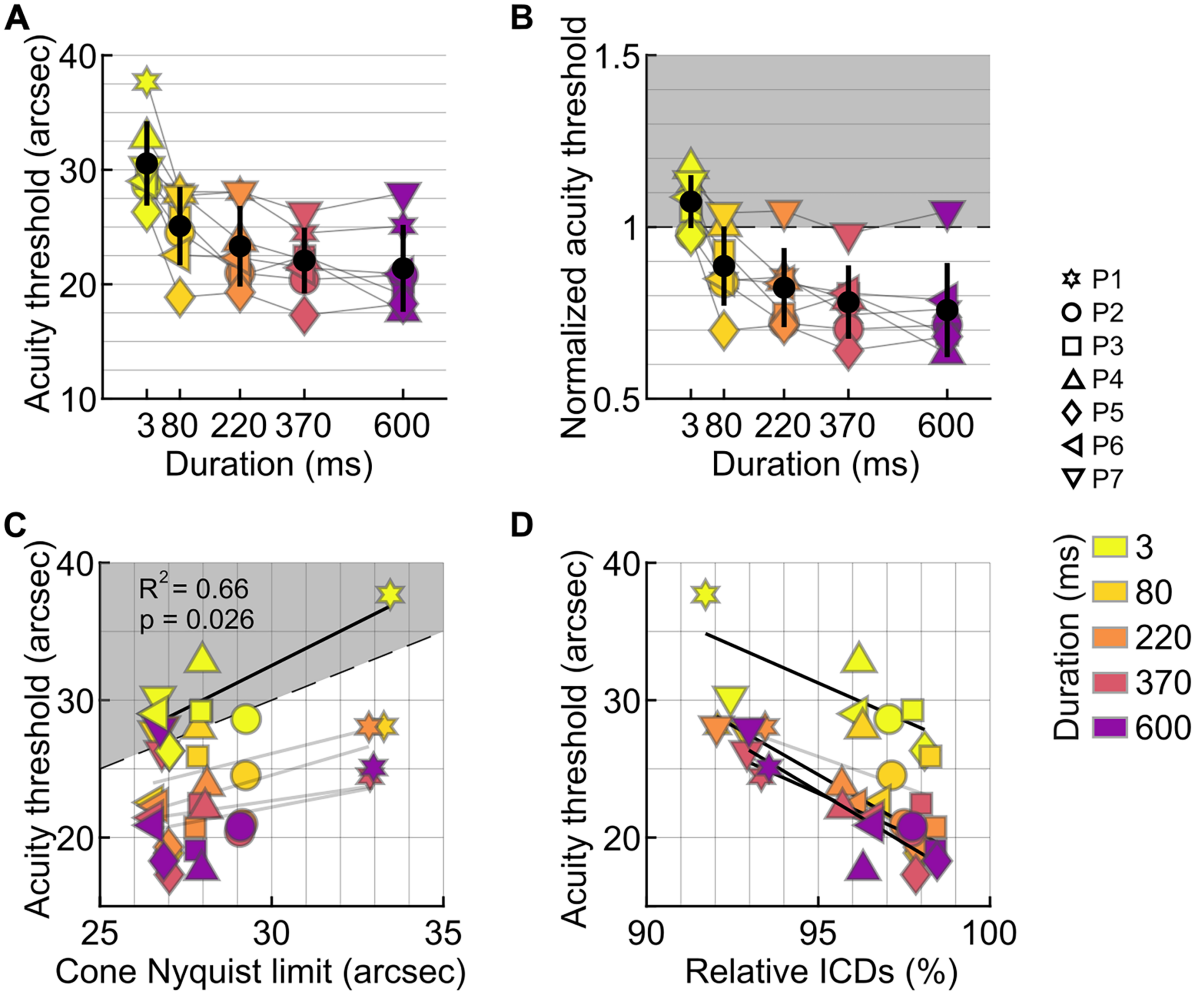
Acuity at different stimulus durations. (**A**) Absolute acuity thresholds as a function of presentation duration in all participants. Black markers are the average and standard deviation across participants per condition. (**B**) Acuity threshold normalized to the individual cone Nyquist limit. The gray area indicates acuity values above the Nyquist limit (normalized values > 1), corresponding to performance worse than the cone resolution limit. (**C**) Acuity as a function of the cone sampling limit. Linear regression was only significant for the flash condition (3 ms). (**D**) Acuity as a function of the ratio between ICDs of seeing cones and ICDs at the CDC.

Acuity improved with increasing presentation duration in all participants, but performance changed abruptly between the ultra-brief 3-ms condition and 80 ms (Figure 5A). At 3 ms, thresholds clustered close to each observer's cone-sampling limit, and normalization to the individual Nyquist limit reduced interparticipant variability (Figure 5B), indicating that cone topography constrained performance when essentially no retinal slip occurred. At 80 ms, thresholds for most participants fell below their cone Nyquist limits, and they continued to improve with longer durations. Beyond this transition, cone density no longer predicted threshold differences. For the three longest durations, thresholds settled into a narrow range across observers, corresponding to roughly 0.8 to 0.75 of the Nyquist limit. The selective reduction in variability at 3 ms (Figures 5A, 5B) and the significant correlation at this duration (*R*^2^ = 0.66, *p* = 0.026; Figure 5C) reinforce that cone sampling was limiting only at zero drift.

Across participants, thresholds improved when areas of higher cone densities were used (Figure 5D). When the ratio between the average ICD across trials in a given condition and the ICD at the CDC was calculated, we found a significant relationship between threshold and such ratio for the 3-ms (*R*^2^ = 0.57, *p* = 0.05), 220-ms (*R*^2^ = 0.9, *p* = 0.0004), 370-ms (*R*^2^ = 0.7, *p* = 0.02), and 600-ms (*R*^2^ = 0.77, *p* = 0.009) durations. For the 80-ms presentation duration, the relationship was not significant (*p* = 0.13), although the trend was similar.

## Discussion

Using adaptive optics micro-psychophysics combined with simultaneous in vivo imaging of the moving human retina, we measured Tumbling-E acuity at varying stimulus durations. We found that sub-Nyquist acuity can be reached at durations as short as 80 ms, corresponding to a retinal slip of less than 1 arcmin, about two foveal cone diameters.

### Cone mosaic limits performance in the absence of FEM

The Nyquist limit represents a theoretical upper bound of visual resolution and has long been considered a fundamental constraint on visual acuity (Westheimer, 1975; Strasburger, Huber, & Rose, 2018). By varying stimulus presentation duration, we found that acuity thresholds closely matched the individual Nyquist limit (Figure 5B) only when the stimulus remained effectively stationary on the retina (3 ms). In the absence of retinal slip, the density of the retinal sampling array must exceed that of the stimulus to allow unaliased signal reconstruction. Otherwise, undersampling may introduce perceptual distortions (but see Ruderman & Bialek, 1992) that mask the true form.

We found that individual retinal sampling limits were surpassed once stimuli were allowed to move on the retina, except in one participant (P7, who seemed an outlier; see below) (Figure 5B). These results confirmed previous findings obtained under optimal optical conditions using AOSLO (Rossi et al., 2007; Witten et al., 2024). Earlier observations with optics-independent interference stimulation showed that line patterns remained detectable up to 90 to 100 cycles/deg, corresponding to a spacing of 18 to 20 arcsec (Williams, 1985). Such thresholds and our observed sub-Nyquist acuities are likely achievable through a dynamic de-alias, where fixational eye movements engage multiple photoreceptors over time and effectively remove artifacts that are due to undersampling.

### Minimal retinal slip has the highest impact on threshold

We found that when there was stimuli slip on the retina, thresholds decreased (i.e., acuity improved). The most pronounced improvement was observed between the 3- and 80-ms presentation durations, with a threshold decrease by 17.5%, on average. At 80 ms, stimulus slip was less than 1 arcmin, on average, less than two foveal cone diameters (Figure 4A). This finding suggests that even minimal retinal slip can enhance spatial sampling by shifting the stimulus across adjacent cones within a short time window. This is in line with earlier behavioral reports (Kuang et al., 2012; Intoy & Rucci, 2020) and theoretical modeling (Pitkow et al., 2007; Ahissar & Arieli, 2012; Nghiem et al., 2025), demonstrating that a retinal slip imposed by FEM aids acuity. The necessary slip amplitudes have been shown to be minimal and do not necessarily have to be stemming from an individual's own eye motion to aid acuity (Ratnam et al., 2017).

When stimulus slip amplitudes are modulated by varying the duration that the stimulus is visible, temporal information integration must be taken into account. According to Bloch's law, longer exposures, even at the same intensity, improve the detectability of small stimuli (Gorea, 2015). A prominent mechanism thought to be at play here is probability summation, where multiple weak or subthreshold signals are combined to increase signal strength (Watson, 1979). Temporal integration was shown to improve performance only with short presentation durations, up to a critical duration reported between 50 and 100 ms (Barlow, 1958; Saunders, 1975; Gorea, 2015), after which partial summation persists up to 650 ms (Holmes, Victora, Wang, & Kwiat, 2017). Similar results emerged in our study, where threshold improvement rates declined beyond ∼80 ms.

In our experiments, performance continued to improve as the duration of stimulus presentation increased (Figures 5A, 5B). However, the relative gains between successive durations decreased and were not statistically significant, with improvements of 7%, 5.4%, and 3% for the longer durations, compared to an improvement of 17.5% from 3 to 80 ms. Prior studies reinforce the notion of a nonlinear temporal integration process, where most perceptual benefits are achieved within the first ∼100 ms of stimulus visibility (Tulunay-Keesey & Jones, 1976; Ng & Westheimer, 2002), after which acuity increases with a gradually decreasing rate (Baron & Westheimer, 1973; Alexander et al., 1993; Niwa & Tokoro, 1997; Heinrich et al., 2010; McAnany, 2014). Our findings align with the principle of nonlinear temporal integration with continued temporal summation (Holmes et al., 2017) in the visual system.

### Interactions between cone topography and FEM

We observed that the benefit of a denser cone mosaic was no longer visible at longer stimulus durations (Figure 5C). At these time scales, neither cone topography nor movement statistics alone predicted acuity. Instead, a more complex picture emerged in which cone topography, retinal location, and movement patterns interacted.

One key aspect of this interaction was the relationship between drift direction and cone topography: Stimuli that were displaced toward regions of higher cone density were associated with better acuity (Figure 5D). This implies that participants often relied on retinal regions with lower cone density than their theoretical maximum, providing, on average, ∼95% of their available cone sampling capacity. Indeed, the centroid of seeing cones was consistently offset from the anatomical center by 1 to 7.2 arcmin across participants. Those with the largest offsets (7.2 and 4.8 arcmin in P1 and P7, respectively) also exhibited the highest thresholds. Such offset fixation appears to be a typical feature of human vision, occurring symmetrically across eyes, but it remains unclear if it serves a particular role (Putnam et al., 2005; Wilk et al., 2017; Wang et al., 2019; Kilpeläinen, Putnam, Ratnam, & Roorda, 2020; Reiniger et al., 2021).

The displacement of the stimulation centroid was further linked to drift velocity (*R*^2^ = 0.74, *p* = 0.01) and drift length at long durations (*R*^2^ = 0.67, *p* = 0.025). These findings indicate that relatively large fixational eye movements can reduce acuity, consistent with previous reports (Clark et al., 2022). Higher FEM velocities tended to shift gaze toward more peripheral retinal regions, where cone size increases and spatial resolution decreases (Rossi & Roorda, 2010; Intoy & Rucci, 2020), even when the displacements occurred within the foveola (Jenks, Carrasco, & Poletti, 2025). As a consequence, performance differences could also reflect a reduced ganglion cell–to–cone ratio in the foveola. Although a uniform 2:1 ratio is often assumed (Curcio & Allen, 1990; McCann, Hayhoe, & Geisler, 2011; Watson, 2014), recent work suggests higher values and greater variability (Drasdo, Millican, Katholi, & Curcio, 2007). Together with emerging evidence of idiosyncratic foveolar cone topography and their functional employment (Reiniger et al., 2021; Witten et al. 2024; Ameln et al., 2025) and asymmetries in visual performance (Jenks et al., 2025), these results point to the need for finer distinctions within foveolar circuitry.

Individual participants further illustrate how FEMs can either aid or harm performance. P7, for example, exhibited fast movements over high-density regions and rarely reached sub-Nyquist acuity (Figure 5B). Having also the highest number of uniquely activated cones, this combination may exceed optimal cone signals (Pitkow et al., 2007; Burak et al., 2010). In contrast, participant P1, with lower baseline cone sampling but equally high drift velocity and the highest drift variance, was able to reach sub-Nyquist thresholds, although their stimulus centroid remained displaced from the densest regions. Interestingly, at 600 ms, both P1 and P7 recruited similar numbers of cones across stimulus sizes between 24 and 36 arcsec, despite P7 having 35% higher cone density at the CDC (Figure 4C). Thus, FEMs acted as an equalizing factor: For P1, they may have enhanced performance by engaging more cones, while for P7, they may have limited the benefit of higher local density. Finally, participants showed consistent and habitual use of retinal areas that display consistent ICDs, evidenced by the low variability in seeing cone densities across conditions (Figure 4C). Similarly, a stable proportion of cones (67%–78%) was recruited throughout trials (Figure 3B). Together with the stereotypical drift patterns observed in P1, P2, and P3 (Figure 3C), these findings suggest that individuals develop characteristic, habitual drift strategies, which may supersede optimal, trial-by-trial trajectories.

## Conclusions

In this study, we investigated how stimulus duration, fixational eye movements, and local cone topography jointly impact foveal visual acuity. We found that visual acuity benefits from naturally occurring fixational eye movements within the first 80 ms of viewing, during which the stimulus is displaced by less than 1 arcmin, corresponding to only about two diameters of the smallest foveal cones. Remarkably, this minimal retinal slip was already sufficient to improve performance from the static, cone-limited regime to sub-Nyquist acuity levels. Beyond this initial interval, acuity continued to improve with increasing stimulus duration, albeit at a reduced rate.

Fixational drift characteristics varied substantially across participants, and their impact on acuity depended on how individual drift trajectories interacted with local cone topography and retinal location. At longer stimulus durations, neither cone density nor drift magnitude alone predicted performance; rather, acuity reflected the combined effect of where stimuli landed, how they moved relative to regions of higher or lower cone density, and the individual's habitual drift strategy. Consistent with this interplay, observers with denser mosaics often drifted across smaller retinal regions, which led to a reduction in the spread of engaged cones across participants.

Together, our findings show that fixational eye movements can both aid and limit visual acuity depending on the time scale. Most importantly, they demonstrate that the visual system can extract meaningful spatial information from extremely small retinal displacements, highlighting the critical role of even minimal drift in shaping foveal vision over brief viewing intervals.
